# The Effect of Material Heterogeneity and Temperature on Impact Toughness and Fracture Resistance of SA-387 Gr. 91 Welded Joints

**DOI:** 10.3390/ma15051854

**Published:** 2022-03-02

**Authors:** Milivoje Jovanović, Ivica Čamagić, Simon Sedmak, Aleksandar Sedmak, Zijah Burzić

**Affiliations:** 1 Department for Mechanics, Faculty of Technical Sciences, University of Priština Temporarily Settled in Kosovska Mitrovica, 38220 Kosovska Mitrovica, Serbia; milivoje.s.jovanovic@gmail.com (M.J.); ivica.camagic@pr.ac.rs (I.Č.); 2 Innovation Center of the Faculty of Mechanical Engineering, 11000 Belgrade, Serbia; simon.sedmak@yahoo.com; 3 Faculty of Mechanical Engineering, University of Belgrade, 11000 Belgrade, Serbia; 4 Military Technical Institute, 11000 Belgrade, Serbia; zijah.burzic@vti.vs.rs

**Keywords:** welded joint, crack-initiation energy, crack-propagation energy, fracture toughness

## Abstract

This paper presents the analysis of the behavior of welded joints made of 9–12% Cr-Mo steel SA-387 Gr. 91. The successful application of this steel depends not only on the base metal’s (BM) properties but even more on heat-affected-zone (HAZ) and weld metal (WM), both at room and at operating temperature. Impact testing of specimens with a notch in BM, HAZ, and WM was performed on a Charpy instrumented pendulum to enable the separation of the total energy in crack-initiation and crack-propagation energy. Fracture toughness was also determined for all three zones, applying standard procedure at both temperatures. Results are analyzed to obtain a deep insight into steel SA 387 Gr. 91’s crack resistance properties at room and operating temperatures. Results are also compared with results obtained previously for A-387 Gr. B to assess the effect of an increased content of Chromium.

## 1. Introduction

The Cr-Mo steel SA-387 Gr. 91 belongs to a group of heat-and creep-resistant 9–12% Cr steels. They are introduced into practice to replace 2.25% Cr-Mo steel for operating temperatures above 565 °C, with the maximum service temperature of Gr. 91 equaling circa 600 °C [1].

The Cr-Mo steel SA-387 Gr. 91 has exceptional mechanical properties, including crack initiation and propagation resistance, making it an excellent choice for pressure vessels operating at elevated temperatures. It is a simple matter to compare SA-387 Gr. 91, in its role as a base metal, with more conventional steels, such as SA-387 Gr. B, and to find out that conventional design methods will lead to significant reductions in pressure vessel thickness and costs in general [1]. However, having in mind the importance and complexity of welded joints, it is of utmost importance to obtain a deep insight into the behavior of all zones (base metal, weld metal, heat-affected zone) to ensure the safe application and exploitation of Cr-Mo steel. Knowing that welded joint crack sensitivity increases, the more complex the composition and structure of a steel becomes, it is reasonable to assume that a complete overview of SA 387 Gr. 91 application must include a detailed analysis of its crack resistance in all welded joint zones. This should include at least Charpy toughness and fracture toughness testing, both at room and elevated temperatures, up to 575 °C, which is the aim of this research.

In a limited number of papers published about the effect of material heterogeneity and temperature on steel SA-387 Gr. 91’s behavior, most of the focus was on strength and creep properties in relation to their microstructure, especially in a HAZ [2,3,4,5,6,7,8,9,10,11]. Detailed study of the simulated heat-affected zone of creep-resistant 9–12% advanced chromium steel is presented in [2,3,4,5], with an emphasis on the microscopic analysis of the influence of multiple thermal cycles on simulated HAZ toughness [3], the relationship between microstructure and mechanical properties [4], and the significance of cracks [5]. The fracture properties of different microstructural regions of the heat-affected zone (HAZ) of modified 9Cr-1Mo steel (tempered base metal, inter-critical, fine grained, coarse grained with and without δ-ferrite) have been also studied using the Charpy impact test in [6]. A simulation technique is used to reproduce HAZ microstructures. The results indicated the lowest toughness in coarse-grained regions of the HAZ [6].

One of the most important problems with SA-387 Gr. 91 is its narrow heat-affected zone with heterogeneous structures in the base metal, generated due to non-equilibrium phase transformations during the arc-welding processes [7,8,9]. This heterogeneous heat-affected zone has been reported to cause the short-term creep failures of welded components, such as the infamous Type IV cracking [6]. It was shown in [7,8,9] that the Post-Weld Heat Treatment (PWHT) plays a significant role in improving weldments’ toughness and maximizing their creep lifetime. A similar study was made on somewhat different steel, Gr 92 and G92N, with a focus on the effect of Boron and Nitrogen, as presented in [10].

The creep-crack growth behavior of a P92 steel-welded joint was analyzed in [11] with the crack tip located at different distinct zones of welded joint. Tested results revealed that even in thin thickness specimens, fine-grained heat-affected zone specimens exhibited a fast creep-crack growth rate compared with other micro-zone specimens due to a low creep crack resistance and a high multi-stress state.

The approach used in this paper had already been applied in the case of structural, low-alloyed HSLA steel and its welded joint constituents [12], as well as in a series of papers presenting research on a similar steel, A 387 Gr. B, with 1% Cr, which is also used for elevated temperature [13,14,15,16,17,18]. In general, all relevant mechanical properties of steel A 387 Gr. B, such as tensile properties, Charpy impact toughness, fracture toughness, and Paris law coefficients, have been presented in series of papers [13,14,15,16,17,18,19], including the effects of time and temperature. More concretely, the influence of temperature and exploitation period (time) on the behavior of a welded joint subjected to impact loading was analyzed in [13], while the same effects on plane strain fracture toughness in a welded joint were analyzed in [14], indicating very good crack resistance properties of all three regions, with small differences between them. One interesting approach to measuring the relationship between the impact and fracture toughness of A-387 Gr. B welded joint was presented in [15], where separated energies as obtained using Charpy instrumented pendulum were compared with fracture toughness. The effect of temperature and exploitation time on tensile properties and plane strain fracture toughness in a welded joint was analyzed in [16,17], also indicating the good properties of all three zones, i.e., BM, WM, and HAZ. The influence of temperature and exploitation period on fatigue-crack growth parameters in different regions of welded joints was analyzed in [18], indicating the highest crack-growth-rate values in HAZ and the lowest in BM. Therefore, the lowest fatigue-crack resistance of steel A-387 Gr. B is in HAZ. Moreover, higher temperatures and longer exploitation periods increase crack growth rates and decrease fatigue thresholds for both new and exploited materials in all regions of welded joints (BM, WM, HAZ). These effects occur as the result of microstructural changes, such as carbide formation and growth at grain boundaries and inside grains [18]. Finally, a recently published paper [19], dealt with the crack resistance of SA 387 Gr. 91 welded joints under static and impact load, presenting preliminary results for Charpy impact toughness and fracture toughness, indicating good resistance to crack growth for all three welded joint regions, but with significant differences between them. As expected, crack resistance in HAZ is reduced compared to BM, and, somewhat less expected, WM is significantly more sensitive to cracking but still performs at a satisfying level. In this paper, more results for Charpy impact toughness and fracture toughness of SA 387 Gr. 91 welded joints and more detailed analysis will be presented, with a focus on material heterogeneity and temperature effects, as already briefly outlined in [19], but also with a focus on the Chromium effect, which was not previously analyzed in this way. Therefore, the results presented here will not be only analyzed on their own, but also in comparison with corresponding results for A-387 Gr. B to obtain a better insight into the effect of a significantly increased content level of Chromium. In any case, the focus in this research is on the effect of weldment heterogeneity (i.e., the different properties of BM, WM, and HAZ) and Cr content on the crack resistance of a welded joint.

## 2. Methods

Steel SA-387 Gr. 91 is designed to have the minimum yield stress of 450 MPa and minimum impact energy of 41 J at room temperature, with the idea that is will be able to work at elevated temperatures with sufficient strength and toughness [1,19]. For this research, steel SA-387 Gr. 91, thickness 15 mm, produced in “Steelwork ACRONI” Jesenice Slovenia, was used, with the chemical composition shown in Table 1.

**Figure 1:**
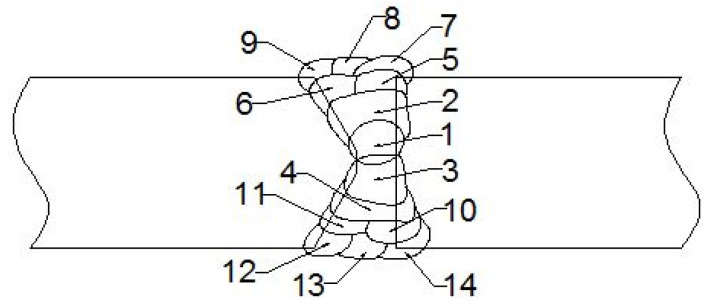
Welded plate (thickness 15 mm) cross-section, welding passes: root 1–4, filler 5–14.

Welding was performed in 4 root and 10 filler passes, using a Gas Tungsten Arc Welding (GTAW) with BOEHLER C9 MV-IG Ø2.4 mm filler metal to ensure high quality of root passes 1–4 and a Shielded Metal Arc Welding (SMAW) with BOEHLER FOX C9 MV electrode, diameters 2.5 (passes 5–9) and 3.25 mm (passes 10–14), as shown schematically in Figure 1. The chemical compositions and mechanical properties of filler metals are shown in Table 2. Welding parameters and linear energies (as shown in Table 3) were chosen carefully to adjust cooling speed and optimize welded-joint microstructure, with thermal efficiency coefficients taken as 0.6 (GTAW) and 0.8 (SMAW).

Pre-heating was performed at 250 °C, while the inter-pass temperature was 200–300 °C. Post-Weld Heat Treatment (PWHT) was applied, consisting of tempering at 250 °C, followed by heating up to 750 °C (rate 100–150 °C/h), holding at 750 for 2 h, and cooling down to 400 °C (rate 150 °C/h), with final cooling at the still air.

### 2.1. Impact Testing on Charpy Instrumented Pendulum

**Figure 2:**
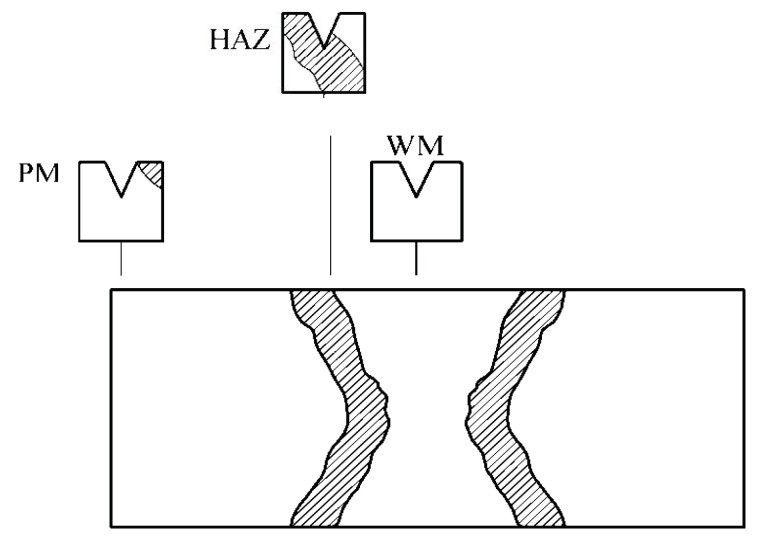
Welded joint Charpy specimen cutting scheme.

The testing procedure was applied according to SRPS EN ISO 9016:2013 [20], including specimen shape and size, as well as notch V-2 position, Figure 2. Testing was performed on an instrumented Charpy pendulum SCHENCK TREBELL 150/300 J at room temperature, 20 °C, and an elevated temperature, 575 °C. The higher temperature was chosen as it is a common service temperature for this steel, whereas room temperature was used as a reference, so that the effect of high temperature on the steel could be evaluated. Three specimens were extracted from each characteristic zone with the crack tip positioned in the BM, WM, and HAZ. In the case of the BM, the specimens were taken from a location far from the weld metal, as a common practice to avoid welding heat effects. In the case of the HAZ, the specimen tip was located in the HAZ, as close to the WM as possible (as shown in Figure 2), since the CGHAZ was shown to have the lowest toughness in HAZ [6]. In the case of the WM specimen, the tip was located close to the center line.

**Figure 3:**
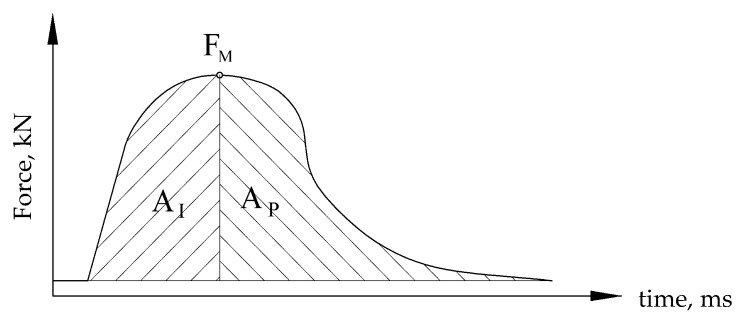
Separation of energies for crack initiation and propagation.

Since the tests were performed using an instrumented Charpy pendulum, it was possible to separate crack initiation and propagation energies, and to evaluate the effect of the notch location on the impact properties and plasticity. In this way, it was possible to determine the energy required for initiating a crack and the energy required for its propagation, enabling better understanding of the crack resistance of tested material, as explained in more details in [21], including different methods to separate these two energies. In this research, separation was performed according to the force maximum value, so that the area to the left represents the energy for crack initiation, A_i_, the area to the right the energy for crack propagation, A_p_, Figure 3.

### 2.2. Fracture Toughness, K_Ic_, Testing

**Figure 4:**
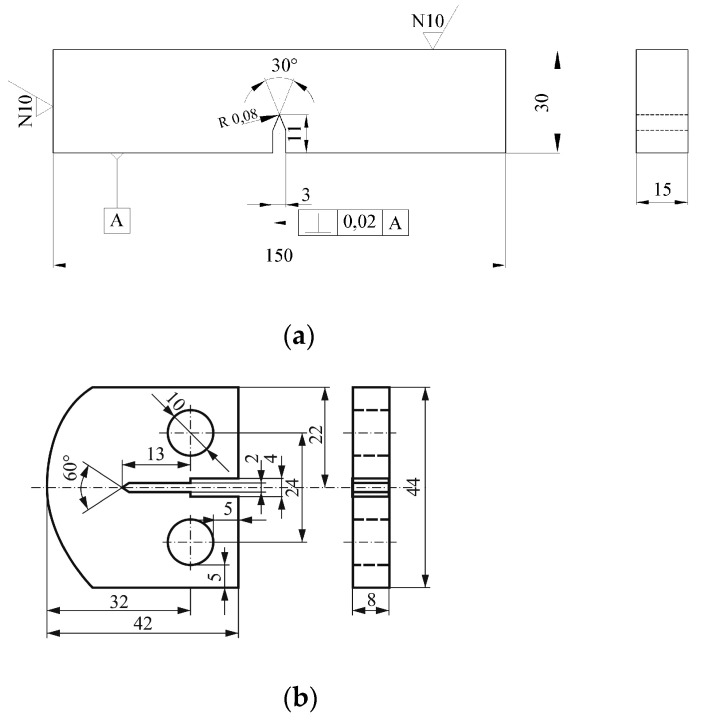
Specimens for *K_Ic_* testing. (**a**) SENB, (**b**) CT.

Three-point single-edge bending (SEB) specimens were used for fracture toughness, *K_Ic_*, measurement at room temperature, whereas modified CT specimens were used at elevated temperature, 575 °C (as shown in Figure 4). This modification was needed due to the shape of the chamber used for testing at 575 °C and had no effect on fracture toughness values, since the stress–strain state at the crack tip was not affected. Fracture toughness, *K_Ic_*, was determined via critical *J* integral, *J_Ic_*, applying rules of elastic–plastic fracture mechanics (EPFM) [22]:

(1) $$K_{Ic}=\sqrt{\frac{J_{Ic}\cdot E}{1-\nu^{2}}},$$

where *E* is the Elasticity modulus, and is *ν* the Poisson ratio. Standard procedure is defined in the ASTM 1820 standard [23] along with the specific aspects for a welded joint testing set out in [24]. A crack was produced on the HF testing machine, and its length was measured after the experiment, as defined in [23].

## 3. Results

### 3.1. Impact Testing

**Figure 5:**
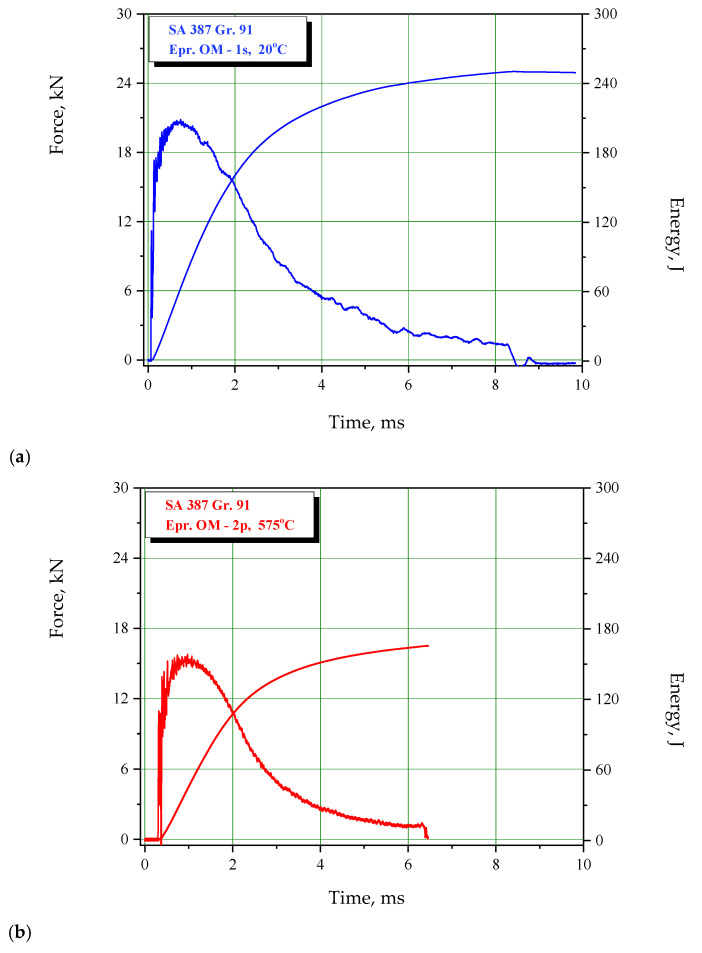
Charpy instrumented pendulum diagrams for BM, SA-387 Gr. 91 at (**a**) 20 °C, (**b**) 575 °C.

**Figure 6:**
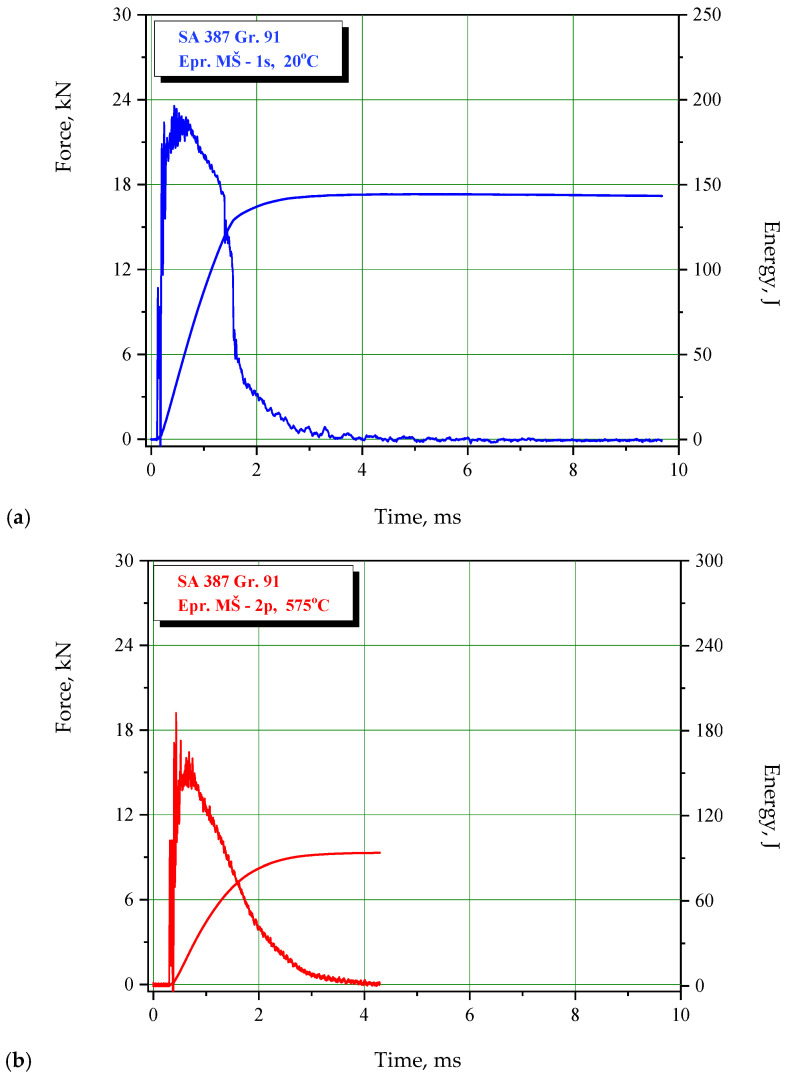
Charpy instrumented pendulum diagrams for WM, SA-387 Gr. 91 at (**a**) 20 °C, (**b**) 575 °C.

**Figure 7:**
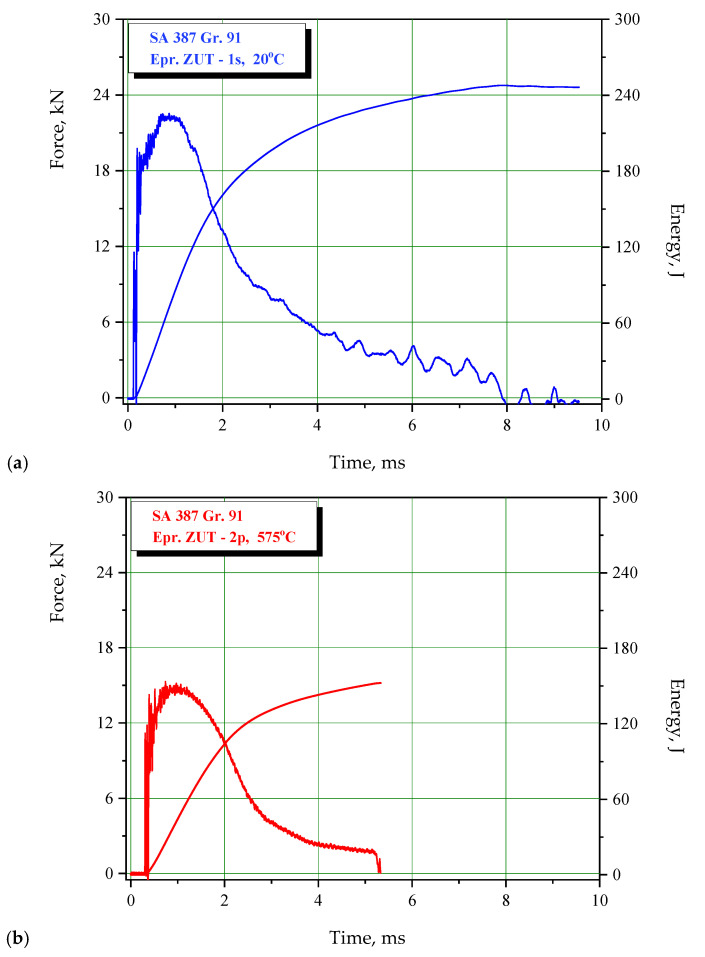
Charpy instrumented pendulum diagrams for HAZ, SA-387 Gr. 91 at (**a**) 20 °C, (**b**) 575 °C.

Results of the impact tests are provided for BM, WM, and HAZ in Table 4, Table 5 and Table 6, respectively. Characteristic examples of F-t diagrams are shown in Figure 5, Figure 6 and Figure 7 for BM, WM, and HAZ, respectively. As one can see from the results presented in Table 4, Table 5 and Table 6, impact toughness at room temperature is the highest in BM, closely followed by HAZ. High resistance to cracking in HAZ is even more pronounced when energy components are considered, since it has the highest resistance to crack initiation. In any case, one should keep in mind that all zones in SA 387 Gr. 91 have a relatively high impact energy, both for crack initiation and propagation, making their welded joints resistant to cracking. At this point, one should notice that such result actually leads to the conclusion that the welding procedure specification for SA 387 Gr. 91 is well defined, and welding itself is well performed.

The results of the impact testing are in good agreement with the presented microstructures and hardness values, since the highest impact energy (BM) corresponds with the lowest hardness, and the lowest impact energy (WM) corresponds with the highest hardness.

Testing at the operating temperature indicates similar behavior, since the reduction of energies is similar: BM 29–43%, WM 26–47%, and HAZ 9–43%. Therefore, energy values at the operating temperature, compared with the room temperature are as follows: BM 57–71%, WM 53–74%, HAZ 57–91%, which are still relatively high. The lowest value is energy for crack initiation, A_I_ = 28 J, which was recorded in WM and is still reasonable from a practical point of view.

**Figure 8:**
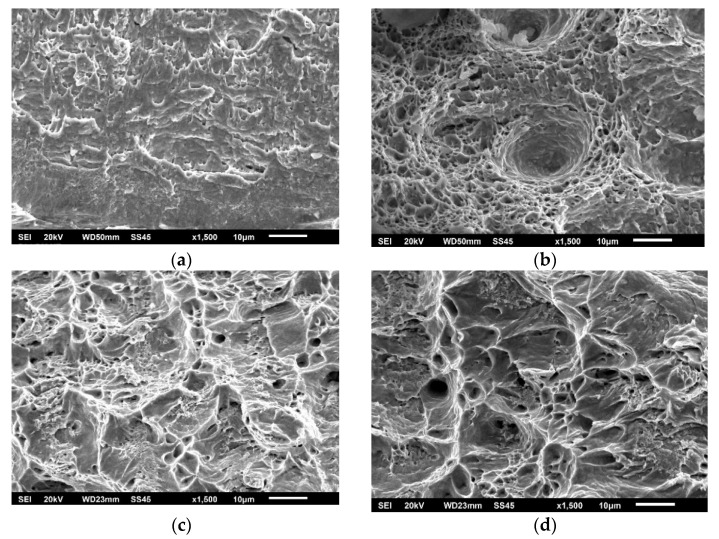
SA-387 Gr. 91 BM fractography upon (**a**) crack initiation, 575 °C; (**b**) crack propagation, 575 °C; (**c**) crack initiation, 20 °C; and (**d**) crack propagation, 20 °C.

**Figure 9:**
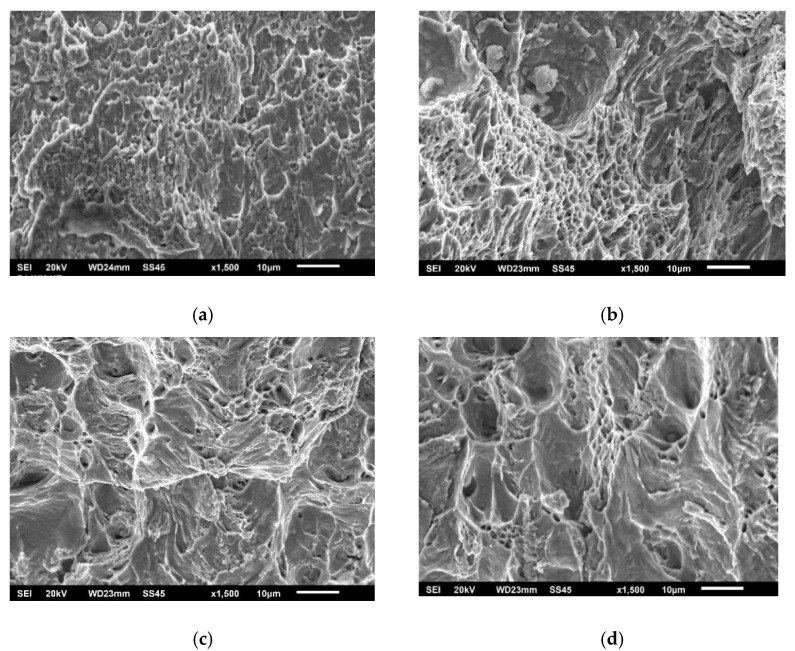
SA-387 Gr. 91 WM fractography upon (**a**) crack initiation, 575 °C; (**b**) crack propagation, 575 °C; (**c**) crack initiation, 20 °C; and (**d**) crack propagation, 20 °C.

**Figure 10:**
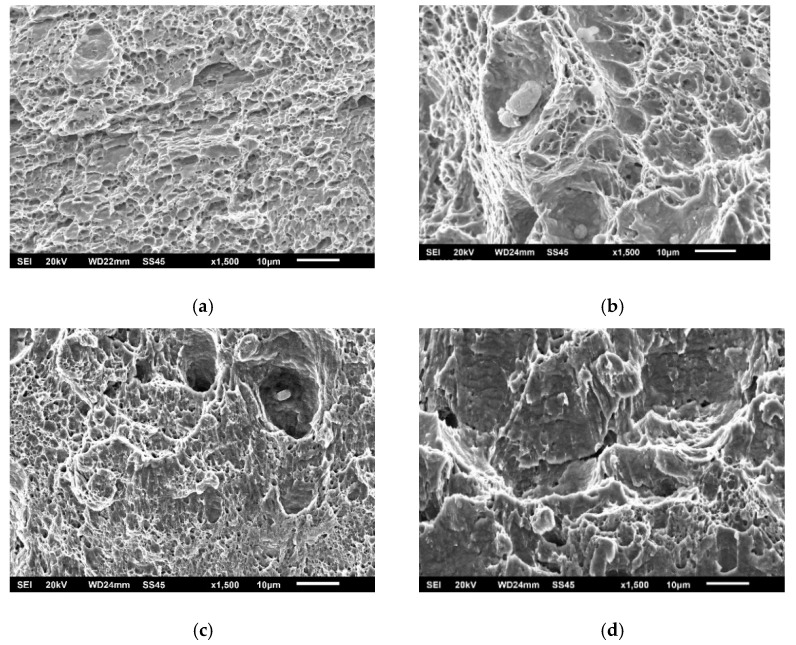
SA-387 Gr. 91 HAZ fractography upon (**a**) crack initiation, 575 °C; (**b**) crack propagation, 575 °C; (**c**) crack initiation, 20 °C; and (**d**) crack propagation, 20 °C.

The effect of different zones in a welded joint on crack initiation and propagation is also visible in Figure 8, Figure 9 and Figure 10, where fractographies of BM, WM, and HAZ are shown, respectively, for both testing temperatures. It is clear that only Figure 8a, Figure 9a and Figure 10a, which present the crack initiation process at 575 °C, do not show completely ductile fracture surfaces, which is in agreement with the lower energies recorded for crack initiation in these specimens (42, 28, and 40 J, respectively). However, they do not represent brittle fractures either; all fractographies indicate sufficient toughness values and high resistance to crack initiation and propagation. Moreover, one should notice relatively small differences in crack initiation and propagation energies between the different zones in a welded joint made of SA387 Gr. 91, which is also proved by the presented fractographies.

The results of the impact testing for A387 Gr. B, obtained at room temperature, are presented in Table 7, Table 8 and Table 9 for BM, WM, and HAZ, respectively. The distribution of energies, both total and separated, is similar as for 9% Cr steel, the highest values are in BM, but are followed closely by both HAZ and WM, in this case. These results also lead to the conclusion that the welding procedure specification for A 387 Gr. B is well defined, and the welding itself is well performed.

The reduction of energy at operating temperature is smaller for steel with 1% Cr than for steel with 9% Cr, with similar distribution of energy: BM 18–38%, WM 8–30%, HAZ 20–29%. The levels of energy in relation to the room temperature are: BM 62–82%, WM 70–92%, HAZ 71–80%. The lowest individual value for initial energy, A_I_ = 38 J, is recorded in BM and is still satisfactory. Nevertheless, one should not forget that the operating temperature for 1% Cr steel is 540 °C, i.e., lower than that for 9% Cr (575 °C), so the reduction of energies was expected, not only because of the simpler microstructure (less Cr).

### 3.2. Fracture Toughness Testing

**Figure 11:**
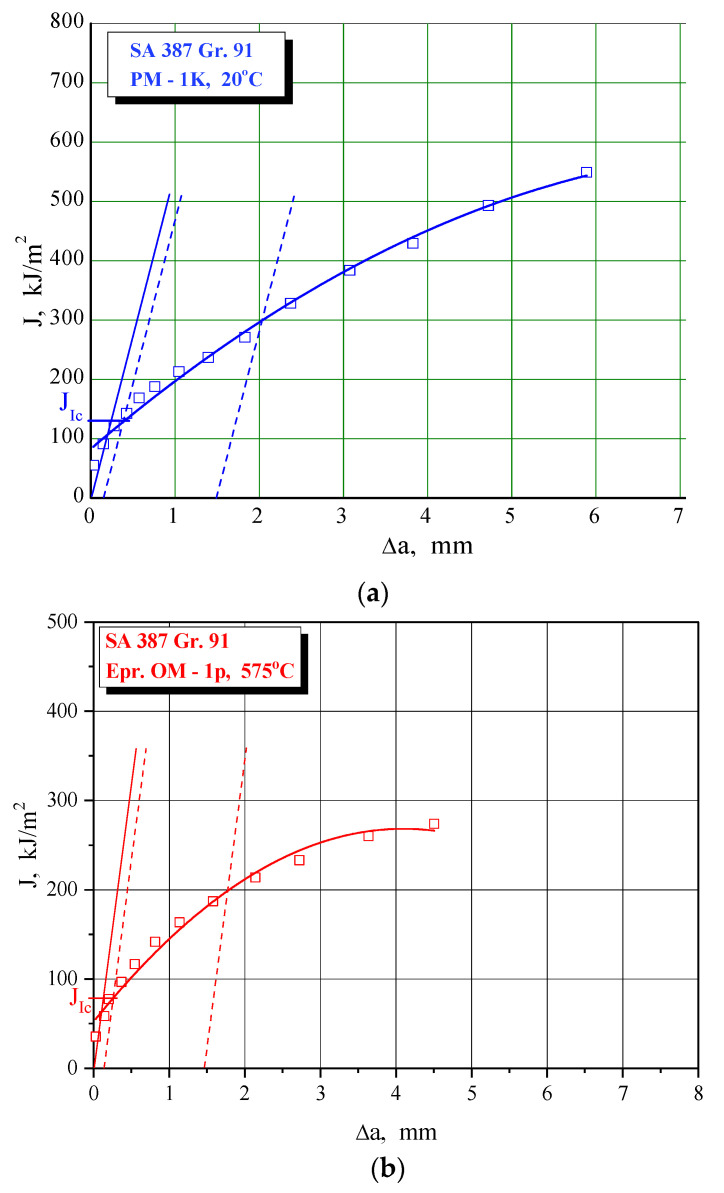
The J-R curve and J_Ic_ evaluation for SA 387 Gr. 9—BM at (**a**) 20 °C, (**b**) 575 °C.

**Figure 12:**
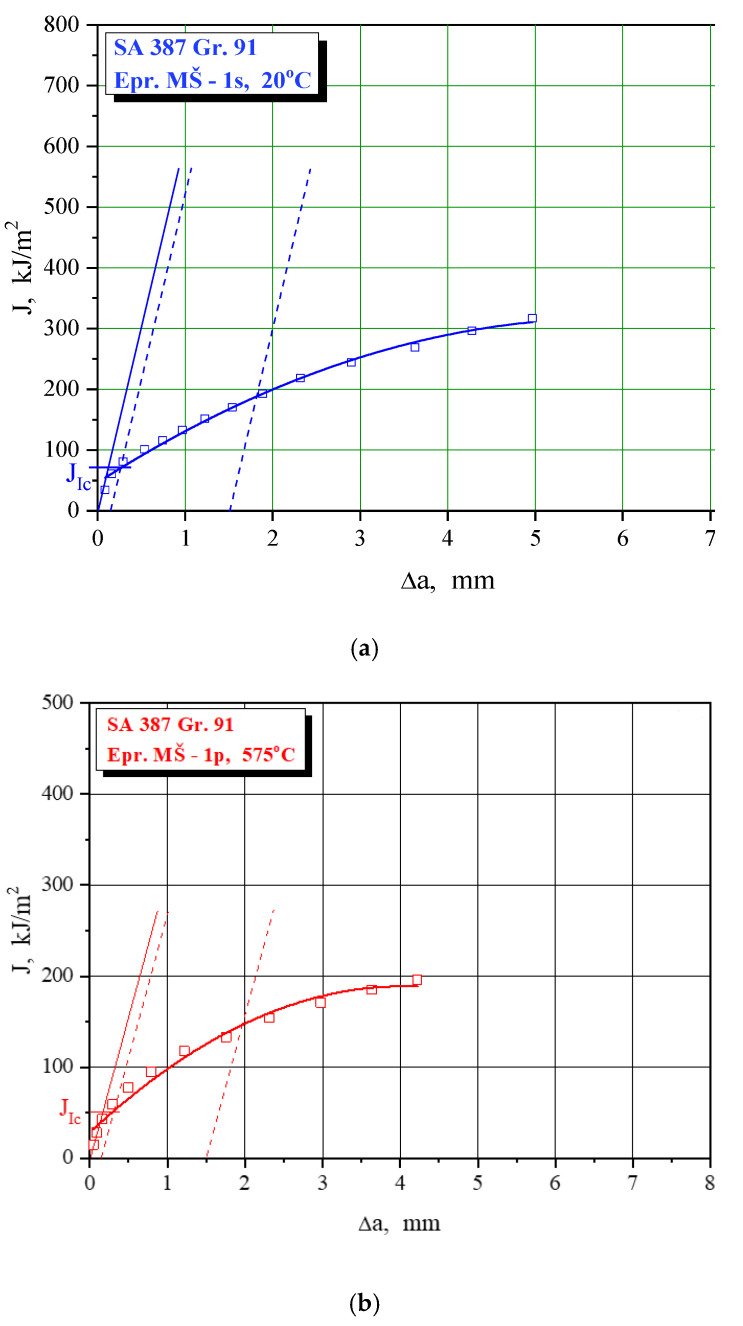
The J-R curve and J_Ic_ evaluation for SA 387 Gr. 91—WM at (**a**) 20 °C, (**b**) 575 °C.

**Figure 13:**
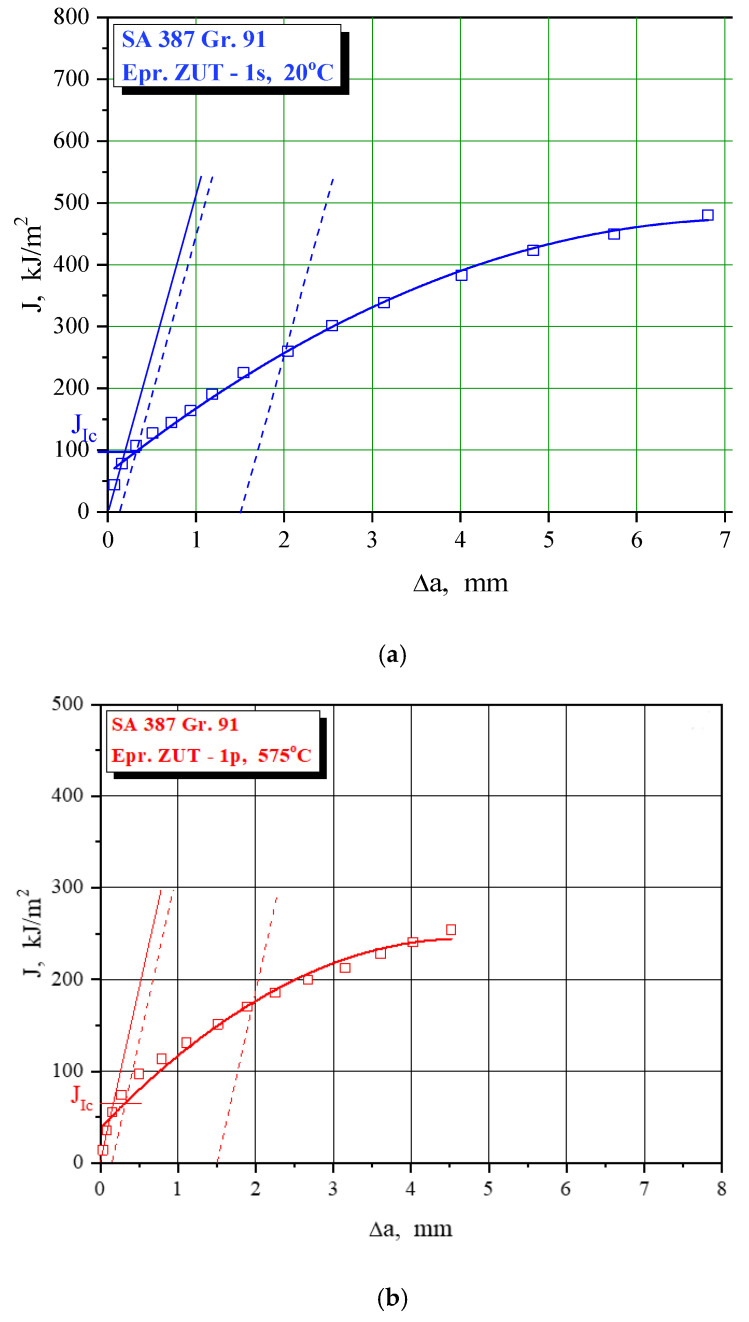
The J-R curve and J_Ic_ evaluation for SA 387 Gr. 91—HAZ at (**a**) 20 °C, (**b**) 575 °C.

Fracture toughness values are obtained via J_Ic_, as explained in Section 2.2, using J-R curves as shown in Figure 11, Figure 12 and Figure 13 for characteristic examples of BM, WM, and HAZ testing, respectively. Calculated K_Ic_ values for SA 387 Gr. 91 steel are provided in Table 10, Table 11 and Table 12 for BM, WM, and HAZ, respectively, clearly indicating that the K_Ic_ values are satisfactory, with the highest values in BM (175.0 and 124.4 J for the room and operating temperature, respectively), the lowest in WM (125.7 and 91.1 J), and in-between in HAZ (146.4 and 111.9 J). The effect of heterogeneity and temperature is similar, as in the case of impact toughness, with HAZ being somewhat more sensitive to cracking, and with a slightly smaller reduction of K_Ic_ values (the ratio between 20 and 575 °C values is circa 1.3 compared to circa 1.6 for impact toughness) with increased temperature.

**Figure 14:**
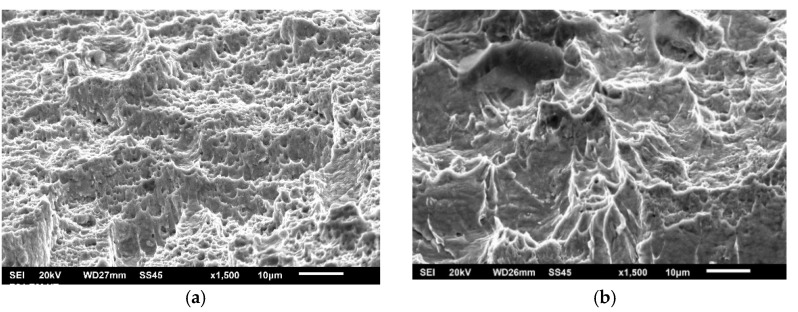
SA-387 Gr. 91 BM fractography. (**a**) SENB specimen 20 °C, (**b**) CT specimen 575 °C, 1500×.

**Figure 15:**
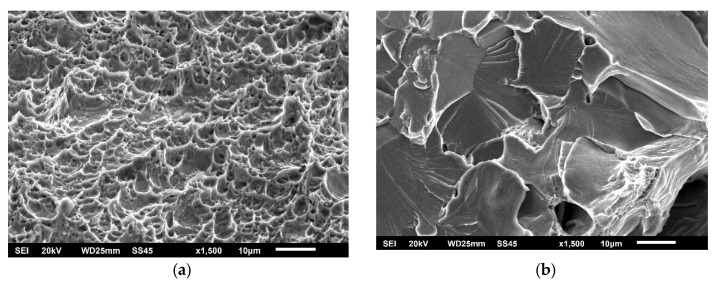
SA-387 Gr. 91 WM fractography. (**a**) SENB specimen 20 °C, (**b**) CT specimen 575 °C, 1500×.

**Figure 16:**
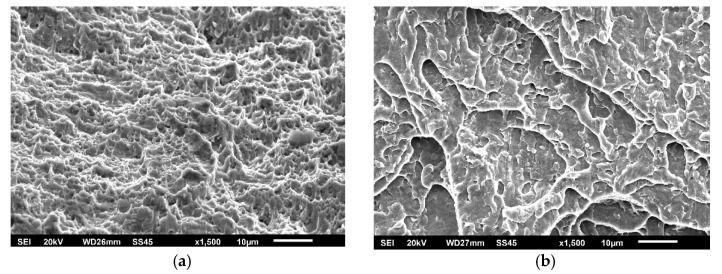
SA-387 Gr. 91 HAZ fractography. (**a**) SENB specimen 20 °C, (**b**) CT specimen 575 °C, 1500×.

**Figure 17:**
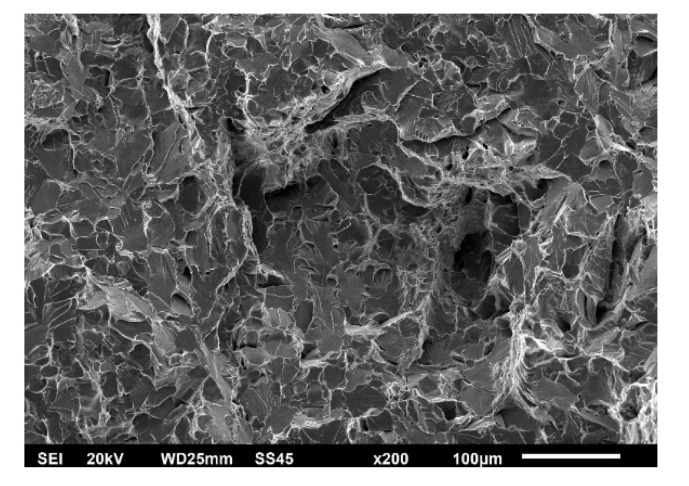
SA-387 Gr. 91 WM fractography, CT specimen 575 °C, 200×.

As in the case of impact toughness, one should notice relatively small differences in K_Ic_ values between the different zones in welded joints made of SA387 Gr. 91, proved also by the presented fractographies (as shown in Figure 14, Figure 15 and Figure 16), indicating sufficiently ductile material. Even in the case of WM at 575 °C (as shown in Figure 15b), which appears to be a brittle fracture, it was found that this is actually a ‘local brittle zone’ (LBZ), not uncommon for WM, especially if made of alloyed steel. The same fractography, but with a magnification of 200 ×, is shown in Figure 17, also indicating some typical features of a ductile fracture.

Values for K_Ic_ in the case of A387 Gr. B steel are provided for comparison in Table 13, Table 14 and Table 15 for BM, WM, and HAZ, respectively. In this case, the results for fracture toughness are different from those for impact toughness in two important aspects: the first is that 1% Cr steel has lower values than 9% Cr, and the second is that WM is now the region with the highest crack resistance, whereas HAZ is the weakest link. However, the differences are very small, since the average values for WM are circa 10% higher, and for HAZ circa 10% lower, than BM average values. Therefore, the effect of heterogeneity is less pronounced than in the case of SA 387 Gr. 91 steel, whereas the effect of temperature is slightly stronger (the ratio between 20 °C and 575 °C values is circa 1.4, which is almost the same as in the case of SA 387 Gr. 91).

## 4. Conclusions

Based on the results presented in this paper, one can conclude the following:

- Both steels, SA-387 Gr. 91 and A-387 Gr. B, as well as their welded joints, have high resistance to cracking, both for static and impact loading. This conclusion also holds for SA 387 Gr. 91 WM, even though its resistance to cracking is lower than BM and HAZ, but well above 41 J, which is the minimum value for the BM.
- The effect of material heterogeneity on impact toughness is more heavily expressed for SA-387 Gr. 91 than for A-387 Gr. B, since the WM in the former case has lower values of crack initiation and growth energies, whereas these values are balanced in the latter case. A reduction of impact toughness in the case of SA-387 Gr. 91 steel is mostly due to crack-growth energy, which is significantly smaller than for SA-387 Gr. 91 BM and HAZ, but still at a satisfying level.
- The effect of temperature on impact toughness is similar, but more pronounced, since both energies are lower in all cases, approximately 1/3 less than at room temperature, but still at a satisfying level.
- The effect of material heterogeneity on fracture toughness is similar to its effect on impact toughness, but more expressed for SA-387 Gr. 91 than for A-387 Gr. B, for the same reason as in the case of impact toughness. The effect of temperature on fracture toughness is also similar to its effect on impact toughness. One can say that the behavior of both materials and their welded joints in respect to cracking is practically the same for static and impact loading.

## Figures and Tables

**Table 1:** Base metal chemical composition, steel SA-387 Gr. 91.

Chemical Composition, Weight %										
C	Si	Mn	P	S	Cr	Mo	Ni	V	Nb	Cu
0.129	0.277	0.443	0.001	0.001	8.25	0.874	0.01	0.198	0.056	0.068

**Table 2:** Filler metal chemical composition (%).

Filler Metal	C	Si	Mn	P	S	Cr	Mo	Ni	V	Nb	Cu
C9 MV-IG Ø2.4 mm	0.11	0.23	0.5	0.006	0.003	9.0	0.93	0.5	0.19	0.07	0.0
FOX C9 MV Ø2.5 mm	0.09	0.19	0.55	0.01	0.006	8.5	1.0	0.5	0.19	0.04	0.1
FOX C9 MV Ø3.25 mm	0.11	0.26	0.66	0.008	0.005	8.5	0.94	0.5	0.20	0.06	0.1

**Table 3:** Welding parameters and linear energies.

Pass	Voltage V	Current A	Welding Speed mm/s	Linear Energy kJ/mm
1	12.2	172	0.3	4.2
2	12.2	172	0.6	2.1
3–4	12.2	172	0.9	1.4
5–9	25.4	126	3.0	0.85
10–14	25.4	126	2.6	0.98

**Table 4:** Results of Charpy testing—SA 387 Gr. 91 BM.

Specimen	Testing Temperature, °C	Impact Total Energy, A_T_, J	Crack-Initiation Energy, A_I_, J	Crack-Growth Energy, A_P_, J
BM-1A		251	58	193
BM-2A	20	268	60	208
BM-3A		275	58	217
average		265	59	206
BM-4A		159	41	118
BM-5A	575	166	43	123
BM-6A		155	41	114
average		160	42	118

**Table 5:** Results of Charpy testing—SA 387 Gr. 91 WM.

Specimen Mark	Testing Temperature, °C	Impact Total Energy, AT, J	Crack-Initiation Energy, AI, J	Crack-Growth Energy, AP, J
WM-1A		144	52	92
WM-2A	20	168	55	113
WM-3A		156	52	104
average		156	53	103
WM-4A		92	28	64
WM-5A	575	94	28	66
WM-6A		104	29	75
average		97	28	69

**Table 6:** Results of Charpy testing—SA 387 Gr. 91 HAZ.

Specimen Mark	Testing Temperature, °C	Impact Total Energy, A_T_, J	Crack-Initiation Energy, A_I_, J	Crack-Growth Energy, A_P_, J
HAZ-1A		248	70	178
HAZ-2A	20	246	69	177
HAZ-3A		248	70	178
average		248	70	178
HAZ-4A		147	39	108
HAZ-5A	575	153	42	111
HAZ-6A		138	40	98
average		146	40	106

**Table 7:** Results of impact testing for A387 Gr. B—BM [13].

Specimen Mark	Testing Temperature, °C	Impact Total Energy, A_T_, J	Crack-Initiation Energy, A_I_, J	Crack-Propagation Energy, A_P_, J
BM-1-1n	20	204	47	157
BM-1-2n		212	49	163
BM-1-3n		214	49	165
average		210	48	162
BM-2-1n	540	137	38	99
BM-2-2n		139	40	99
BM-2-3n		145	41	104
average		141	40	101

**Table 8:** Results of impact testing for A387 Gr. B/WM [13].

Specimen Mark	Testing Temperature, °C	Impact Total Energy, A_T_, J	Crack-Initiation Energy, A_I_, J	Crack-Propagation Energy, A_P_, J
WM-1-1	20	193	56	137
WM-1-2		190	60	130
WM-1-3		183	60	123
average		189	59	130
WM-2-1	540	139	40	99
WM-2-2		133	39	94
WM-2-3		134	39	95
average		135	39	96

**Table 9:** Results of impact testing for A387 Gr. B—HAZ [13].

Specimen Mark	Testing Temperature, °C	Impact Total Energy, A_T_, J	Crack-Initiation Energy, A_I_, J	Crack-Propagation Energy, A_P_, J
HAZ-1-1e	20	186	47	139
HAZ-1-2e		187	45	142
HAZ-1-3e		183	47	136
average		185	46	139
HAZ-2-1e	540	143	46	97
HAZ-2-2e		131	43	88
HAZ-2-3e		129	42	87
average		134	44	90

**Table 10:** K_Ic_ values obtained via J_Ic_ for SA 387 Gr. 91—BM.

Specimen Mark	Testing Temperature, °C	Critical J-Integral, J_Ic_, kJ/m^2^	Critical Stress Intensity Factor, K_Ic_, MPa∙m^1/2^
BM-1K	20	131.1	173.9
BM-2K		144.2	182.4
BM-3K		124.0	169.2
average			175.0
BM-4K	575	78.5	122.9
BM-5K		80.9	124.7
BM-6K		81.9	125.5
average			124.4

**Table 11:** K_Ic_ values obtained via J_Ic_ for SA 387 Gr. 91—WM.

Specimen Mark	Testing Temperature, °C	Critical J-Integral, J_Ic_, kJ/m^2^	Critical Stress Intensity Factor, K_Ic_, MPa∙m^1/2^
WM-1K	20	71.6	128.5
WM-2K		64.8	122.3
WM-3K		69.2	126.4
average			125.7
WM-4K	575	51.2	99.2
WM-5K		40.1	87.8
WM-6K		38.6	86.2
average			91.1

**Table 12:** K_Ic_ values obtained via J_Ic_ for SA 387 Gr. 91—HAZ.

Specimen Mark	Testing Temperature, °C	Critical J-Integral, J_Ic_, kJ/m^2^	Critical Stress Intensity Factor, K_Ic_, MPa∙m^1/2^
HAZ-1K	20	97.6	150.1
HAZ-2K		88.9	143.2
HAZ-3K		92.1	145.8
average			146.4
HAZ-4K	575	65.3	112.1
HAZ-5K		61.6	108.8
HAZ-6K		68.5	114.8
average			111.9

**Table 13:** Values of K_Ic_ via J_Ic_ for A387 Gr. B—BM.

Specimen Mark	Testing Temperature, °C	Critical J-Integral, J_Ic_, kJ/m^2^	Critical Stress Intensity Factor, K_Ic_, MPa∙m^1/2^
BM-1-1n	20	60.1	117.8
BM-1-2n		63.9	121.4
BM-1-3n		58.6	116.3
average			118.5
BM-2-1n	540	43.2	87.2
BM-2-2n		44.7	88.7
BM-2-3n		45.3	89.2
average			85.7

**Table 14:** Values of K_Ic_ via J_Ic_ for A387 Gr. B—WM.

Specimen Mark	Testing Temperature, °C	Critical J-Integral, J_Ic_, kJ/m^2^	Critical Stress Intensity Factor, K_Ic_, MPa∙m^1/2^
WM-1-1	20	72.8	129.6
WM-1-2		74.3	130.9
WM-1-3		71.1	128.1
average			129.5
WM-2-1	540	50.2	93.9
WM-2-2		52.6	96.2
WM-2-3		48.4	92.2
average			94.1

**Table 15:** Values of K_Ic_ via J_Ic_ for A387 Gr. B—HAZ.

Specimen Mark	Testing Temperature, °C	Critical J-Integral, J_Ic_, kJ/m^2^	Critical Stress Intensity Factor, K_Ic_, MPa∙m^1/2^
HAZ-1-1n	20	53.6	111.2
HAZ-1-2n		51.7	109.2
HAZ-1-3n		49.8	107.2
average			109.2
HAZ-2-1n	540	33.6	76.9
HAZ-2-2n		34.2	77.5
HAZ-2-3n		36.1	79.7
average			78.0
